# Perioperative local anesthetics as modulators of tumor progression and metastasis

**DOI:** 10.3389/fphar.2026.1793731

**Published:** 2026-04-22

**Authors:** Xiaoyu Zheng, Huaying Wei

**Affiliations:** Department of Surgical Anesthesiology, Shengzhou People’s Hospital (Shengzhou Branch of the First Affiliated Hospital of Zhejiang University School of Medicine), Shengzhou, Zhejiang, China

**Keywords:** cancer progression, immune modulation, local anesthetics, opioid-sparing analgesia, perioperative anesthesia, tumor metastasis, voltage-gated sodium channels

## Abstract

Cancer surgery remains a cornerstone of treatment for solid malignancies; however, perioperative factors increasingly emerge as critical modulators of tumor recurrence and metastasis. Accumulating evidence suggests that anesthesia techniques and analgesic strategies can profoundly influence tumor biology, immune competence, and long-term oncologic outcomes. In particular, opioids—while indispensable for perioperative analgesia—have been associated with enhanced tumor progression through direct stimulation of oncogenic signaling pathways and suppression of antitumor immunity. In contrast, regional anesthesia and local anesthetics have gained attention for their opioid-sparing properties and potential antitumor effects. Beyond effective analgesia, local anesthetics exert multifaceted actions on tumor progression by inhibiting voltage-gated sodium channels, attenuating inflammatory signaling, suppressing EGFR-driven oncogenic pathways, restraining mesenchymal stem cell–mediated tumor support, and preserving or enhancing antitumor immune responses. This review summarizes current preclinical and clinical evidence elucidating the mechanisms by which local anesthetics and regional anesthesia modulate tumor growth, metastasis, and immune surveillance. Understanding these perioperative interactions provides a compelling rationale for integrating anesthetic strategies into comprehensive oncologic care aimed at improving long-term cancer outcomes.

## 1 Introduction

Cancer remains a leading cause of morbidity and mortality worldwide, posing a growing threat to public health amid global population aging (GBD, 2025). Although substantial progress has been made in clinical treatment strategies for solid tumors, surgical resection remains the cornerstone of therapy (Onuma et al., 2020; Chen et al., 2025). Nevertheless, tumor recurrence and metastasis following surgery continue to represent major therapeutic challenges. Increasing evidence suggests that perioperative factors, particularly anesthesia techniques and agents, may significantly influence tumor biology, including metastatic potential, recurrence rates, and long-term prognosis (Snyder and Greenberg, 2010; Heaney and Buggy, 2012).

Regional anesthesia has gained traction not only for its efficacy in perioperative pain control and opioid-sparing effects but also for its potential impact on tumor progression (Ses et al., 2019; Weng et al., 2016). By blocking afferent neural input, regional anesthesia may suppress activation of the hypothalamic–pituitary–adrenal (HPA) axis, attenuate systemic inflammation, and mitigate surgery-induced immunosuppression (Desborough, 2000; Kurosawa, 2012). Moreover, local anesthetics have been shown to directly exert antitumor effects, including the inhibition of proliferation, invasion, and migration of cancer cells (Piegeler et al., 2012). This review summarizes recent advances in our understanding of how regional anesthesia and local anesthetics may modulate tumor metastasis and progression, and to explore the underlying mechanisms involved.

## 2 Opioids promote tumor progression

### 2.1 Reducing opioid requirement as a strategy to suppress tumor growth

Tumor metastasis remains a primary cause of cancer-related mortality, making the control of tumor cell spread a critical therapeutic objective (Fonteyne et al., 2026). While opioids are indispensable for managing cancer pain, emerging evidence suggests that their use may be associated with shortened progression-free survival (PFS) and overall survival (OS), implying a potential link between opioid exposure and increased tumor burden (Zylla et al., 2014; Lennon et al., 2012). In orthotopic models of human and murine breast cancer, opioid exposure has been shown to enhance tumor cell migration and activate key signaling pathways, such as STAT3 (Gu et al., 2025; Wu Z. S. et al., 2025; Tripolt et al., 2021). Specifically, the opioid-induced activation of the JAK1/2–STAT3 axis may facilitate epithelial–mesenchymal transition (EMT), thereby promoting cancer metastasis (Tripolt et al., 2021). Furthermore, stimulation of the μ-opioid receptor (MOR) appears to enhance both tumor cell proliferation and metastatic potential (Gorur et al., 2021; Belltall et al., 2022; Bortsov et al., 2012). For instance, morphine activates tumor cell growth via EGFR-mediated signaling, a process amplified by the RhoA pathway to facilitate cancer cell invasion (Yu et al., 2022). Beyond direct tumor stimulation, opioids exert significant immunosuppressive effects, notably by inhibiting T cell proliferation and differentiation (Ninnemann et al., 2024; Min et al., 2025). Even low doses or single administrations can promote tumor growth by suppressing natural killer (NK) cell activity and inducing the expansion of regulatory T cells (Tregs) (Raigon et al., 2021; Ponferrada et al., 2020). Similarly, fentanyl and sufentanil have been demonstrated to decrease NK cell activity while promoting cancer cell migration and invasion (Maher et al., 2019; Won et al., 2023). Therefore, opioids may exacerbate cancer progression through a dual mechanism: direct stimulation of tumor growth and indirect suppression of antitumor immunity, ultimately leading to poorer clinical outcomes (Bortsov et al., 2012; Forget et al., 2019). Consequently, reducing perioperative opioid consumption while maintaining effective analgesia could improve cancer prognosis. Although local anesthetics cannot fully replace opioids, their strategic integration into perioperative care may reduce opioid requirements, thereby indirectly restraining tumor progression (Sun et al., 2012).

### 2.2 Local anesthetics as an opioid-sparing strategy in cancer surgery

Clinical evidence indicates that the administration of local anesthetics can effectively reduce opioid demand in cancer patients. This reduction potentially mitigates opioid-induced tumor-promoting effects, decreases immune suppression, and limits metastatic stimuli, thereby improving overall cancer outcomes. For instance, continuous thoracic epidural analgesia in cardiac surgery patients significantly lowered opioid consumption compared to systemic administration (Pala et al., 2020). Similarly, continuous local anesthetic infusion following mastectomy reduced opioid requirements while maintaining adequate analgesia (AlFaraj et al., 2022). Furthermore, continuous wound infiltration has provided effective pain relief after abdominal and thoracic surgeries, reducing total opioid use without increasing the risk of infection or wound complications (Huang et al., 2021). Researchers have also investigated perioperative analgesic strategies for thoracoscopic lobectomy, finding that combining ropivacaine with dexmedetomidine prolonged postoperative analgesia duration and significantly reduced the need for perioperative fentanyl and sufentanil (Yang J. et al., 2022). Overall, various perioperative local anesthetic techniques can substantially decrease opioid requirements. By alleviating the immunosuppressive and pro-metastatic effects associated with opioids, these strategies not only enhance acute pain control but may also contribute to long-term oncologic benefits.

## 3 Roles of local anesthetics in counteracting tumor progression

### 3.1 Local anesthetics inhibit tumor growth via voltage-gated sodium channels (VGSCs) blockade

Voltage-gated sodium channels (VGSCs), traditionally recognized for their role in initiating and propagating action potentials in excitable tissues, are increasingly implicated in the pathophysiology of malignancy. Aberrant expression of VGSCs has been identified in invasive tumor cells of prostate, cervical, and breast cancers, where they function as critical regulators of cell migration and invasion (Fraser et al., 2014; Wu J. et al., 2025; Liu et al., 2025). VGSCs represent a promising therapeutic target in oncology. Local anesthetics, widely utilized for their sodium channel-blocking properties (Wu et al., 2024; Krogman et al., 2024), offer a mechanistic avenue to attenuate VGSC activity during the perioperative period (Haraguchi-Suzuki et al., 2025). This suppression reduces the ability of tumor cells to escape from the surgical field and metastasize, thus limiting tumor spread and indirectly prolonging patient survival. Accordingly, targeting VGSCs represents a promising therapeutic avenue in oncology, where local anesthetics may serve as adjunctive agents to suppress tumor growth (Roger et al., 2015; Yang et al., 2017). Amide-type local anesthetics, particularly lidocaine and ropivacaine, have demonstrated potent antineoplastic effects via VGSC inhibition. Experimental evidence indicates that ropivacaine suppresses the metastatic potential of colorectal cancer cells by specifically inhibiting Nav1.5 activity (Koltai, 2015). Similarly, lidocaine has been shown to impede Nav1.5-mediated EMT signaling in human ovarian cancer cells (A2780 and SKOV3), resulting in reduced proliferation, migration, and invasion (Chen et al., 2024; Liu et al., 2021).

### 3.2 Potential impact of regional anesthesia on tumor progression

Regional anesthesia has been associated with a reduced incidence of tumor metastasis and recurrence (Brummelhuis et al., 2021). Evidence suggests that local anesthetics can directly modulate neoplastic behavior, given that elevated VGSC activity is frequently observed across various malignancies (Roger et al., 2015; Yang et al., 2017). Thus, blockade of these channels via local anesthetics may interfere with processes that facilitate tumor progression. For instance, experimental data demonstrate that lidocaine suppresses Nav1.5-mediated epithelial–mesenchymal transition signaling, resulting in diminished migration, invasion, and proliferation of human ovarian cancer A2780 and SKOV3 cells (Liu et al., 2021). Similarly, ropivacaine has been shown to inhibit Nav1.5 channel function and metastatic capability, indicating potential therapeutic benefits during the perioperative period in oncologic resections (Baptista-Hon et al., 2014). Furthermore, compounds including lidocaine, ropivacaine, and bupivacaine impede VGSC activity, thereby reducing tumor cell proliferation and differentiation. These agents also exhibit cytotoxic effects on mesenchymal stem cells (MSCs), which are recognized contributors to tumor growth and dissemination, particularly within the stromal microenvironment (Lucchinetti et al., 2012). In recent years, a range of pharmacological agents targeting sodium channels has been developed as part of antitumor strategies (Liu et al., 2024a). Local anesthetics, acting as sodium channel blockers with inherent analgesic and anti-inflammatory actions, may favorably modulate postoperative immune dynamics (Dunn and Durieux, 2017). These properties support their potential integration into adjunctive oncologic regimens, where they might improve clinical outcomes by limiting tumor expansion and dissemination (Table 1).

**TABLE 1:** Molecular and cellular mechanisms through which local anesthetics suppress tumor progression.

Role	Molecular targets	Biological effects	Function
VGSC blockade	Nav1.5, VGSC signaling	Inhibition of tumor cell migration, invasion, and EMT	Lidocaine and ropivacaine suppress Nav1.5 activity, reducing metastatic potential in colorectal, breast, and ovarian cancer models
Anti-inflammatory effects	TNF-α, IL-6, IL-1β, Src, MAPK	Reduced endothelial barrier disruption and tumor cell transendothelial migration	Local anesthetics attenuate TNF-α–Src–MAPK signaling and preserve endothelial integrity
EGFR pathway inhibition	EGFR, HB-EGF, miR-520a-3p, miR-539	Suppression of tumor proliferation, invasion, and survival	Lidocaine downregulates EGFR signaling directly or via miRNA-mediated mechanisms
EMT suppression	EMT-related transcriptional programs	Reduced metastatic dissemination	Inhibition of Nav1.5 and EGFR signaling limits EMT induction
MSC inhibition	TGF-β signaling, cyclin D1, CDKs, ROS	Suppression of MSC proliferation and tumor-supportive differentiation	Local anesthetics inhibit MSC expansion and CAF-like differentiation
Angiogenesis modulation	VEGF, MMP-9	Reduced neovascularization and stromal remodeling	Decreased pro-angiogenic factor release in perioperative settings

### 3.3 Local anesthetics attenuate inflammatory responses to inhibit tumor cell metastasis

Surgical intervention fundamental to oncological management, the procedure inherently triggers inflammatory processes that disturb the equilibrium between pro- and anti-inflammatory cytokines, frequently culminating in postoperative immunosuppression (Guo et al., 2024; Bezu et al., 2024). Even with minimally invasive procedures, the suppression of pro-inflammatory cytokine expression (IL-1β, IL-6, and TNF-α) and interferon-γ (IFN-γ) responses still fails to prevent local skin incisions from triggering inflammation in patients. Inflammation is characterized by vascular leakage (edema and hyperemia), which damages the endothelial barrier and facilitates tumor cell transendothelial migration (Cai et al., 2023; Shenoy and Lu, 2016). The release of inflammatory cytokines can also stimulate the production of MMP-9 and VEGF in the peritumoral stroma, while activating oncogenic Src signaling, thereby compromising endothelial integrity and promoting tumor cell migration and EMT (Lian et al., 2023; Patel et al., 2016). Local anesthetics have been proposed to exert indirect antineoplastic effects through their anti-inflammatory properties (Chamaraux-Tran and Piegeler, 2017). At perioperative concentrations, lidocaine diminishes Src activation and attenuates MAPK phosphorylation, thereby significantly inhibiting TNF-α-driven pro-tumor signaling and reducing TNF-α expression, ultimately suppressing tumor cell invasion (Piegeler et al., 2015). Similarly, both lidocaine and ropivacaine have been shown to interfere with Src-mediated TNF-α signaling and its downstream consequences, preserve endothelial barrier function, and decrease levels of the adhesion molecule ICAM-1 and neutrophil adhesion (Müller et al., 2021).

In individuals with detectable circulating tumor cells (CTCs), the excision of primary tumors can provoke systemic inflammatory responses that facilitate CTC release, diminish immune surveillance, and enhance metastatic spread (Cheng et al., 2022). While general anesthesia (GA) often induces systemic inflammation and stress reactions that may impair host defense mechanisms, local anesthetics appear to mitigate these stress responses, thereby fostering a more protective perioperative milieu. Amide-type local anesthetics exhibit robust anti-inflammatory properties that have been extensively corroborated in both experimental models and human trials (Reysner et al., 2024). The administration of regional anesthesia during the perioperative period significantly curbs inflammatory reactions and confers advantages in suppressing the release of pro-inflammatory cytokines (Reysner et al., 2024; Hashemian et al., 2025). Agents such as lidocaine, bupivacaine, and ropivacaine have been shown to diminish the secretion of MMP-9 and VEGF, downregulate immune activation, and consequently attenuate surgery-associated inflammation (Weinschenk et al., 2022). Importantly, these anti-inflammatory actions may contribute to the inhibition of tumor cell proliferation, survival, and dissemination, potentially improving long-term patient outcomes (Chida et al., 2024; Salib et al., 2025).

Comparative clinical investigations evaluating GA versus local anesthesia (LA) in patients undergoing radiofrequency ablation (RFA) for HCC have demonstrated that GA elevates circulating inflammatory cytokines and promotes hepatoma cell migration as well as EMT in HepG2 cells, effects that are notably attenuated under LA (Shi et al., 2021; Lai et al., 2012). Lidocaine’s anti-inflammatory and analgesic properties significantly improved gut function, reduced pain, and minimized postoperative nausea and vomiting (PONV), thus shortening recovery time and improving survival in colorectal cancer patients (Ji et al., 2021; Guo et al., 2025). Moreover, in patients undergoing resection of brain tumors, lidocaine administration significantly reduced systemic levels of inflammatory markers such as IL-6 and TNF-α, improved the quality of postoperative recovery, and may offer neuroprotective benefits that mitigate secondary cerebral damage (Zhao et al., 2022; Tseng et al., 2023).

### 3.4 Local anesthetics inhibit EGFR activity and suppress tumor progression

**FIGURE 1:**
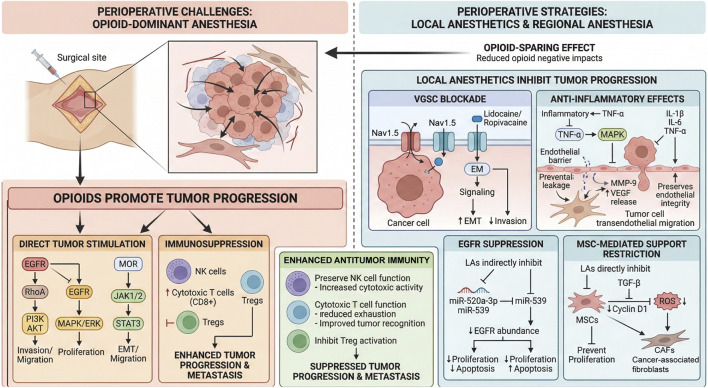
Roles of local anesthetics in tumor progression.

Local anesthetics have been demonstrated to directly modulate the activity of the epidermal growth factor receptor (EGFR), a transmembrane tyrosine kinase receptor whose aberrant activation drives proliferation, invasion, and metastatic dissemination across multiple cancer types (Zhang et al., 2021; Liu et al., 2023). Given its central role in oncogenic signaling, EGFR represents one of the most clinically relevant therapeutic targets in contemporary oncology. Experimental evidence from oral squamous cell carcinoma models revealed that lidocaine suppresses EGFR phosphorylation and downstream signal transduction, resulting in impaired growth of CAL27 cells. In colorectal cancer, lidocaine exposure upregulated miR-520a-3p—a microRNA subject to negative regulation by EGFR. Since EGFR is a validated direct target of miR-520a-3p, its increased expression leads to reduced EGFR abundance, suppression of proliferative capacity in PC and RB cells, and induction of apoptotic cell death (Qu et al., 2018). Complementary investigations demonstrated that lidocaine inhibits the shedding of heparin-binding EGF-like growth factor (HB-EGF), thereby attenuating the invasive phenotype of fibrosarcoma HT1080 cells (Mammoto et al., 2002). In addition, lidocaine upregulates miR-539, which targets EGFR and suppresses its activation (Sun and Sun, 2019). This regulation results in reduced growth and metastatic potential in lung cancer cell lines A549 and NCI-H1299. The inhibitory effect of local anesthetics on EGFR activity may also help reverse MDR and improve the efficacy of chemotherapy. Local anesthetics can suppress EGFR signaling and downregulate P-glycoprotein (Hu et al., 2017) (Figure 1).

### 3.5 Local anesthetics suppress mesenchymal stem cell (MSC)-Mediated tumor support

MSCs are self-renewing, multipotent progenitors of non-hematopoietic tissues that exhibit a pronounced tropism toward tumor sites. Accumulating evidence indicates that MSCs actively contribute to tumor progression through the secretion of pro-tumorigenic growth factors, thereby promoting both tumor expansion and metastatic dissemination (Cammarota and Laukkanen, 2016; Chen et al., 2019). Within the tumor microenvironment, MSCs undergo dynamic phenotypic and functional reprogramming, enabling their integration into tumor-supportive stromal networks. A central mechanism underlying MSC-mediated tumor facilitation is their involvement in EMT, a process through which epithelial cells acquire mesenchymal characteristics associated with enhanced migratory and invasive capacities. During this transition, MSCs can differentiate into cancer-associated fibroblasts (CAFs), a key stromal component that supports tumor cell plasticity, extracellular matrix remodeling, and metastatic spread (Xie et al., 2025; Guo and Xu, 2024; Antoon et al., 2024).

Some reports indicate that MSCs may further potentiate tumor progression by differentiating into pro-angiogenic cell populations characterized by the expression of VEGF, FGF, PDGF, and other angiogenesis-associated mediators, thereby enhancing tumor vascularization and promoting the proliferation of vascular smooth muscle cells (Moradi-Gharibvand and Hashemibeni, 2023). MSCs are also highly responsive to endocrine and signaling cues that govern lineage commitment and pathological reprogramming. For example, parathyroid hormone (PTH) has been shown to induce osteogenic differentiation of rat bone marrow–derived MSCs, while simultaneously stimulating the phosphorylation of GSK3β and β-catenin, ultimately suppressing MSC mineralization and osteogenesis (Liu et al., 2024b). Local anesthetics appear to exert anti-proliferative effects on MSCs through the coordinated modulation of multiple signaling pathways. Mechanistically, these effects have been linked to inhibition of TGF-β signaling, downregulation of the mitogenic regulator cyclin D1, and attenuation of reactive oxygen species (ROS) and other active oxygen intermediates generated in digested cells (Gray et al., 2015). Therefore, local anesthetics suppress MSC proliferation via multiple mechanisms. Given that complete resection of tumors is rarely possible and that surgical injury can cause systemic activation of MSCs, which act as key mediators of tumor growth and metastasis, anesthetic interventions that inhibit MSC activation may provide anti-metastatic benefits (Antoon et al., 2024; Zhang et al., 2023). Indeed, emerging evidence suggests that perioperative administration of local anesthetics may reduce intraoperative tumor dissemination, and their inhibitory effects on MSC proliferation may contribute to decreased metastatic risk and improved long-term outcomes in patients with cancer (Carnet Le Provost et al., 2024). Interestingly, PTH has also been reported to markedly reduce the invasiveness and metastatic capacity of HCC cells, potentially through suppression of hepatocyte growth factor (HGF)-induced activation of c-Met, a mesenchymal-associated marker, together with inhibition of downstream oncogenic signaling cascades. Through this mechanism, PTH may restrain EMT and ultimately reduce tumor metastasis (Yang MH. et al., 2022). Thus, these findings raise the possibility that local anesthetics may not only suppress MSC proliferation, but also interfere more broadly with tumor cell differentiation, tumor initiation, and transcriptional programs associated with metastatic progression.

## 4 Local anesthetics enhance immune cell function to exert antitumor effects

Unlike opioid analgesics, intravenous anesthetics, and inhalational anesthetics, which are generally associated with immunosuppression, local anesthetics may enhance immune cell function (Luan et al., 2022). By maintaining immune system activity, local anesthetics can exert indirect antitumor effects. Whether used alone or in combination with general anesthesia, local anesthetics have been shown to preserve immune cell viability, prolong RFS, and increase overall survival in cancer patients (Bezu et al., 2024; Badwe et al., 2023). Local anesthetics appear to mitigate the perioperative decline in critical effector populations: comparative studies indicate that local anesthetics-based regimens better preserve circulating T lymphocyte and NK cell subsets relative to general anesthesia alone, suggesting superior attenuation of surgery-induced immunosuppression (Cui et al., 2020). In breast cancer patients, the absolute numbers of total leukocytes, lymphocytes, and T cell subsets were higher postoperatively in patients who received local anesthesia than in those who received general anesthesia (Vrbanović Mijatović et al., 2022).

Epidural and intravenous administration of local anesthetics has been associated with improved overall survival in patients with cancer. Mechanistically, these benefits may arise, at least in part, from the capacity of local anesthetics to modulate key immunosuppressive cell populations and restore antitumor immune surveillance. In particular, local anesthetics may inhibit Tregs, which are known to suppress cytotoxic CD8^+^ T cell activity and thereby facilitate tumor progression (Plücker et al., 2021). A substantial body of evidence further indicates that local anesthetics preserve NK cell function, enhance NK cell activity, and reduce CTC levels, although the underlying mechanisms are likely to be multifactorial (Xia et al., 2023). Local injection of local anesthetics into the tumor site in mouse models significantly reduced tumor cell migration and metastasis, possibly via direct activation of NK cells (Bezu et al., 2022). Local anesthetics preserved NK cell function in mouse models of breast cancer metastasis and reduced cancer cell migration to distant organs (Vrbanović Mijatović et al., 2022). Moreover, intravenous lidocaine has been shown to enhance NK cell cytotoxicity by promoting the release of granzyme B and perforin, two key effector molecules involved in tumor cell killing (Lv et al., 2021). In addition to their effects on innate immunity, local anesthetics also appear to potentiate adaptive antitumor responses. Intraoperative administration of ropivacaine reduced the expression of major histocompatibility complex class I (MHC-I) molecules on tumor cells by disrupting lysosomal acidification, thereby enhancing CD8^+^ T cell–mediated recognition and cytotoxic elimination of malignant cells. This effect was accompanied by a marked increase in CD8^+^ T cell infiltration into residual tumor tissues and a reduced likelihood of tumor recurrence (Zhao et al., 2023). In a clinical trial, patients with gastric cancer undergoing laparoscopic radical gastrectomy who received perioperative lidocaine infusion exhibited higher postoperative CD4^+^ T cell levels and increased CD4^+^/CD8^+^ ratios compared with those who did not receive lidocaine, suggesting improved preservation of T cell immunity (Lv et al., 2021). In parallel, perioperative administration of local anesthetics has been reported to enhance cytotoxic T lymphocyte–mediated tumor cell killing while attenuating T cell exhaustion in patients undergoing tumor resection (Carnet Le Provost et al., 2024; Wu Y. Y. et al., 2025). Overall, local anesthetics not only minimize perioperative immunosuppression and preserve immune activity, but also potentially enhance immune cell proliferation, thereby contributing to the antitumor immune response.

## 5 Conclusion

Emerging experimental and clinical data increasingly support the concept that anesthetic management is not a neutral component of cancer surgery but rather an active modulator of tumor biology and host immunity. Opioids, although effective for analgesia, may facilitate tumor progression through activation of oncogenic signaling pathways, promotion of epithelial–mesenchymal transition, and suppression of antitumor immune surveillance. In contrast, regional anesthesia and local anesthetics appear to confer protective effects by reducing opioid exposure and directly interfering with multiple hallmarks of cancer progression. These include inhibition of voltage-gated sodium channels associated with tumor invasiveness, attenuation of surgery-induced inflammatory cascades, suppression of EGFR-mediated proliferative signaling, and restriction of mesenchymal stem cell–driven tumor support. Collectively, these mechanisms suggest that local anesthetics exert both direct and indirect antitumor effects during the vulnerable perioperative window.

Importantly, local anesthetics also demonstrate immunomodulatory properties distinct from those of general and opioid-based anesthesia. By preserving natural killer cell activity, enhancing cytotoxic T lymphocyte function, and limiting regulatory T cell–mediated immunosuppression, local anesthetics may strengthen antitumor immune responses and reduce postoperative metastatic risk. While current evidence is compelling, most data remain derived from preclinical models or retrospective clinical studies. Large-scale, prospective randomized trials are urgently needed to define optimal anesthetic strategies, dosing regimens, and patient populations most likely to benefit. Integrating anesthetic choice into multidisciplinary oncologic decision-making may represent a novel, low-cost, and clinically feasible approach to improving long-term cancer outcomes beyond traditional surgical and systemic therapies.
